# Comparison of radiation doses between diagnostic full‐field digital mammography (FFDM) and digital breast tomosynthesis (DBT): a clinical study

**DOI:** 10.1002/jmrs.405

**Published:** 2020-06-03

**Authors:** Akram M. Asbeutah, Abdullah A. AlMajran, Ajit Brindhaban, Saad A. Asbeutah

**Affiliations:** ^1^ Department of Radiologic Sciences Faculty of Allied Health Sciences Kuwait University Sulaibekhat Kuwait; ^2^ Department of Community Medicine & Behavioral Sciences Faculty of Medicine Kuwait University Sulaibikhat Kuwait; ^3^ Health Sciences Center Faculty of Medicine Kuwait University Sulaibikhat Kuwait

**Keywords:** Breast cancer, digital breast tomosynthesis, full‐field digital mammography, average glandular dose, entrance skin dose

## Abstract

**Introduction:**

There are increasing concerns about radiation exposure among women who undergo full‐field digital mammography (FFDM) and digital breast tomosynthesis (DBT). The main aim of this study was to compare the entrance surface dose (ESD) and average glandular dose (AGD) from FFDM and DBT for different breast thicknesses.

**Methods:**

The ESD and AGD for FFDM in craniocaudal, mediolateral oblique and DBT in craniocaudal projection were recorded from a GE Senographe Essential FFDM unit. The accuracy of the ESD and AGD from the FFDM unit was verified during regular quality assurance programme. Patients were categorised according to their compressed breast thicknesses. X‐ray tube potential and target filter combinations were varied with ESD and AGD recorded directly from the FFDM unit. The non‐parametric Kruskal–Wallis, Mann–Whitney and Wilcoxon signed‐rank tests were performed.

**Results:**

The median and interquartile range (IQR) age of the patients were 48 and 11 years, respectively. The highest median for ESD and median total AGD for different breast thicknesses were ranged from 3.3 to 9.1 mGy and 3.3 to 6.0 mGy, respectively, for two‐view FFDM. However, it ranged from 3.1 to 8.9 mGy and 1.8 to 4.0 mGy, respectively, for single‐view DBT. Both ESD and AGD were significantly lower for DBT (*P* < 0.001) compared with FFDM. There was a significant difference (*P* = 0.001) in the ESD and AGD values for different breast thicknesses in FFDM and DBT techniques.

**Conclusions:**

The AGD for a single‐view DBT was lower than the two‐view FFDM technique.

## Graphical abstract

There are some concerns about increasing the radiation dose among women undergoing both full‐field digital mammography (FFDM) and digital breast tomosynthesis (DBT). In our study, we found that the entrance skin dose (ESD) and total average glandular dose (AGD) are lower in DBT than FFDM techniques. This offers opportunities for the implementation of DBT in the breast cancer detection.

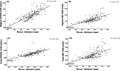

## Introduction

Breast cancer is the most common malignancy in women and one of the leading causes of cancer‐related deaths, and its early detection is important for successful treatment.^1^ Full‐field digital mammography (FFDM) is the most suitable breast imaging technique for diagnostic and/or screening purposes.^2^ Recent advances in DM have led to the introduction of digital breast tomosynthesis (DBT) as an additional and/or alternative technique. DBT is an imaging technique that uses multiple low‐dose projections along an arc over the breast to create thin axial image slices of the breast.^3^ It has significantly improved screening mammography and increased breast cancer detection rate compared to FFDM.^4^ DBT has also reduced the call‐back rates by approximately 30–40%^5^, ^6^ due to improvements in both sensitivity and specificity in diagnostic population compared to FFDM.^6^, ^7^

It has been advocated that DBT should become an integral part of breast cancer screening.^8^ Regardless of whether DBT is used complementary to FFDM or as a replacement, care should be taken concerning the potential increase in the radiation dose delivered to the breast.^9^ This has raised some concerns, among imaging professionals, about increased radiation dose to women undergoing both FFDM and DBT. ^9^ In order to be introduced in screening, where most women do not have breast cancer, radiation dose from DBT should be kept as low as reasonably achievable while maintaining diagnostic accuracy. Radiation dose is cumulative over time, so it is prudent to investigate the level of radiation exposure from DBT.

DBT has been implemented as a diagnostic tool rather than a screening technique for the past six years in our country and for many years in some parts of the world.^9^ A previously published research paper^10^ concluded that the translation of this technology may impact clinically by early detection of a breast lesion and further improvement in patient management, whether used for screening or diagnostic purposes. However, limited work has been published on radiation doses from DBT or a combination of DBT and FFDM for a range of clinically relevant mammographic parameters.^11^, ^12^, ^13^, ^14^ A precise comparison of dose levels between FFDM and DBT, based on clinical data, is still lacking. It is our opinion that sufficient comparison of radiation doses, in terms of entrance surface dose (ESD) and average glandular dose (AGD), for breasts using a subset of women undergoing diagnostic FFDM and DBT is still lacking.

The aim of this study was to explore the relationship between the different mammographic parameters such as compressed breast thickness, exposure factors, and target/filter combination and the radiation dose values from FFDM and DBT techniques.

## Methods

### Subject population and study design

In this prospective study, exposure and dosimetry data for 200 female patients were recorded directly from the mammography unit. Patients were referred to the Breast Imaging Unit in our hospital, following a palpable breast lump on clinical examination or for a second opinion after suspicious findings on ultrasound examination. The DBT examination was acquired at the same time and day following the FFDM. All patients included in this study underwent FFDM and DBT between October 2018 and October 2019. The requirement for obtaining informed consent was waived by the combined Ethical Committee of Health Sciences Centre – Kuwait University, and Ministry of Health, Kuwait, as patient identifying detail was not collected. In order to compare the radiation dose from DBT with radiation dose from FFDM, only patients with complementary DBT and FFDM examinations performed on the same mammography unit were included. The exclusion criteria included women with breast implants, pregnancy, lactation, use of manual exposure factors, no breast compression during imaging and a compressed breast thickness exceeding 8 cm. For analytical simplicity, the patients were divided into six groups according to the compressed breast thickness of 2.1–3, 3.1–4, 4.1–5, 5.1–6, 6.1–7 and 7.1–8 cm.

A total of 400 CC FFDM, 400 MLO FFDM images and 400 CC DBT images were available for analysis. There were 5 (2.5%), 14 (7%), 42 (21%), 80 (40%), 46 (23%) and 13 (6.5%) patients having compressed breast thicknesses of 2.1–3.0, 3.1–4.0, 4.1–5.0, 5.1–6.0, 6.1–7.0 and 7.1–8.0, respectively.

### FFDM and DBT image acquisition

A dedicated Senographe Essential (General Electric Healthcare, Buc, France) mammography unit with Caesium Iodide detectors of 24 × 31 cm, pixel pitch of 100µmm, dual‐track X‐ray tube with Molybdenum/Rhodium (Mo/Rh) target/filter combination, and 5:1 anti‐scatter grid with DM and DBT was used for imaging. The system was subjected to regular quality control programmes concerning technical, dosimetry and image quality aspects. All FFDM and DBT examinations were obtained using conventional setup and fully automatic exposure control (AEC) mode, allowing the unit to determine exposure parameters such as X‐ray tube voltage (kV)/tube current–time product (mAs) combinations and target/filter combinations. The breast compression force was applied at a level depending on pain threshold of each patient. The FFDM images were acquired in craniocaudal (CC) and mediolateral oblique (MLO) projections using the same breast compression.

The DBT procedure included nine projections over an X‐ray tube rotation arc of ±25° from the vertical axis with standard breast compression in CC projection only. DBT image acquisition was performed in a step‐and‐shoot mode, with less than 10 seconds of acquisition time for one breast. Image reconstruction was performed immediately after image acquisition with a slice thickness of 0.5 mm and a reconstruction time of less than 15 seconds. Patient‐related data, such as age, projection orientation (CC or MLO), compressed breast thickness, compression force, exposure factors, target/filter combination, and ESD and AGD, were retrieved directly from the Picture Archiving and Communication System (PACS, General Electric Centricity, version 4.0SP11, USA).

### Radiation dose estimation

The PACS system was used to access images using dedicated high‐resolution mammographic monitors (Barco, 5MP, Belgium). The ESD and AGD values were recorded directly for each FFDM and DBT view, and the radiation dose between the two techniques was compared by per‐view analysis. The reported ESD and AGD values were verified during regular quality assurance (QA) measurements, using conversion coefficients reported in literature.^13^ The QA measurements included ESD measurements at the entrance skin location to verify ESD values reported by the mammography unit routinely. All the images were reported on by two experienced radiologists, as per the general practice in this imaging centre, confirming the diagnostic quality of the images. Also, the radiologist classified the breast composition according to the previously mentioned method.^15^ This method depends on the principle that fat is radiologically lucent and appears dark on a mammogram, whereas connective and epithelial tissues are radiologically dense and appear light. The entire procedure was performed by one technologist and two authors (AA and AB) with more than 20 years of combined experience in breast imaging.

### Statistical analysis

All statistical analyses were performed using Statistical Package for Social Sciences (SPSS) version 25 for Windows (SPSS Inc., Chicago, IL, USA). The ESD and AGD for both FFDM and DBT were analysed for different breast thicknesses, different exposure factors and different target/filter combinations. A Shapiro–Wilk test was performed to test for normality of the variables at *P* = 0.05 level. The non‐parametric Kruskal–Wallis test, followed by pairwise test, and a sub‐analysis with respect to mammographic exposure parameters were performed in case a statistically significant difference in radiation doses between FFDM and DBT techniques. In addition, the Mann–Whitney and Wilcoxon signed‐rank tests were performed to test whether there were any statistically significant differences in radiation doses between the two techniques (DBT‐FFDM). A linear regression test was performed to test whether there was any correlation between the different mammographic parameters. Statistical significance for all tests was considered at *P* < 0.05 level.

## Results

All the breasts were of fibro‐fatty composition. The median and IQR were reported because the Shapiro–Wilk test for ESD and AGD, for both FFDM and DBT techniques, indicated non‐normal distribution (*P* < 0.05) of data. There was no significant difference in age among all groups of different breast thickness (*P* > 0.05). The median, IQR and range of age for all groups were 48, 11 and 33–81 years. Table 1 demonstrates patient age according to breast thickness categories and different target/filter combination.

**Table 1:** Patient characteristics and mammographic parameters for FFDM and DBT techniques for different compressed breast thicknesses.

Breast Thickness Parameter	Technique	2.1–3 cm	3.1–4 cm	4.1–5 cm	5.1–6 cm	6.1–7 cm	7.1–8 cm
Number		5	14	42	80	46	13
Age, Median (IQR), years (Range)		45 (13) (33–52)	46.5 (17.3) (37–71)	49.5 (16.3) (39–81)	48.5 (11) (36–73)	46 (11.3) (38–70)	50 (11) (38–61)
kV, Median (IQR) (Range)	FFDM	27 (0) (27–27)	28 (0.5) (27–29)	29 (0) (27–31)	29 (1) (29–31)	29 (1) (29–31)	30 (1) (29–30)
	DBT	26 (2) (26–29)	29 (0) (29–29)	29 (0) (29–29)	29 (0) (29–31)	29 (2) (29–31)	31 (0) (29–31)
mAs, Median (IQR) (Range)	FFDM	37.7 (10.6) (26.6–43.2)	50.5 (8.3) (39.7–56.3)	48.7 (12.6) (35.3–62.7)	54.1 (7.9) (42.2–96.3)	61.2 (10.4) (49.8–83.0)	71.1 (13.1) (60.8–94.3)
	DBT	4 (1.5) (2.9–4.9)	4.5 (0.3) (3–5)	5.3 (1.2) (3.5–8.6)	6.3 (1.7) (4.2–8.7)	7.8 (2) (5.6–14.1)	7.8 (10.6) (6.5–11.9)
Target/Filter	FFDM	Mo/Rh	Mo/Rh	Rh/Rh	Rh/Rh	Rh/Rh	Rh/Rh
	DBT	Rh/Rh	Rh/Rh	Rh/Rh	Rh/Rh	Rh/Rh	Rh/Rh

DBT = digital breast tomosynthesis; FFDM = full‐field digital mammography; IQR = interquartile range; Mo = Molybdenum; Rh = Rhodium.

### Exposure parameters

**Figure 1:**
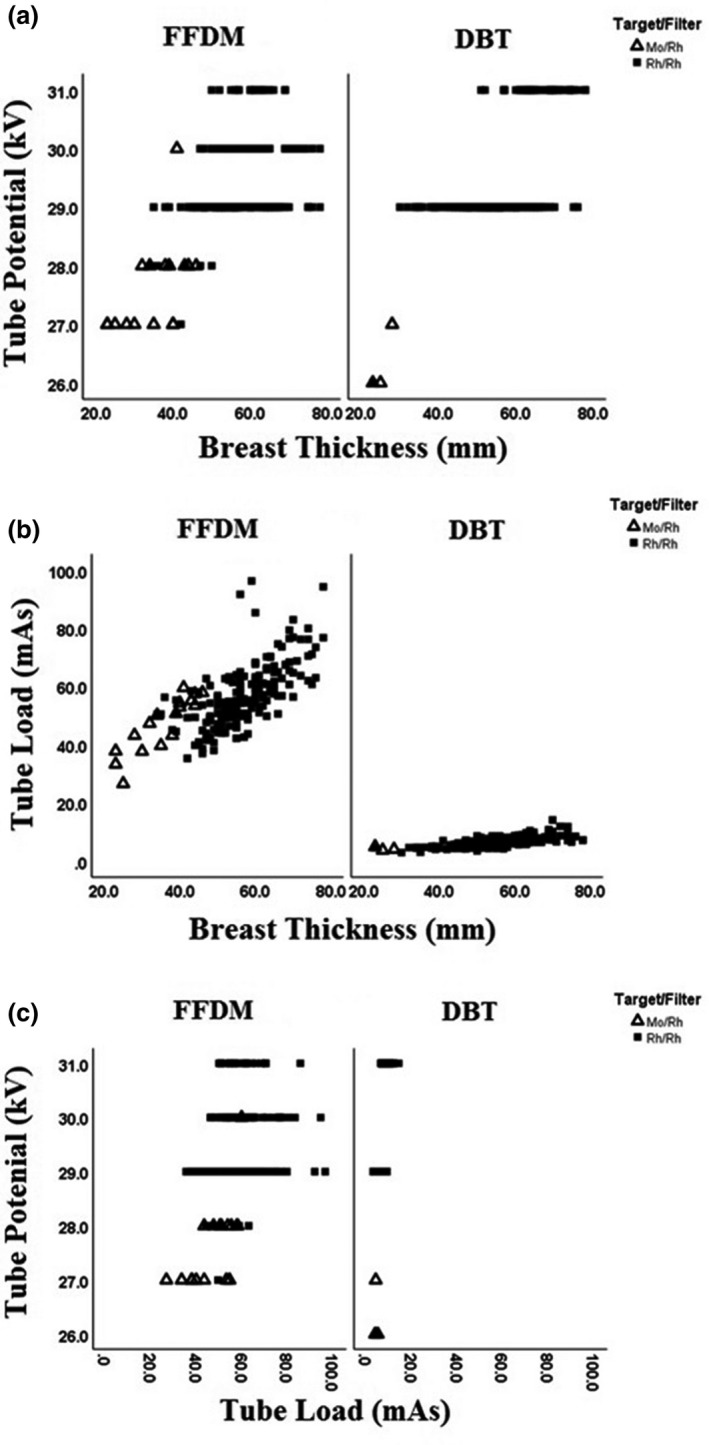
Exposure parameters in individual examinations for full‐field digital mammography (FFDM) and digital breast tomosynthesis (DBT) with different target/filter material (Mo = Molybdenum, Rh = Rhodium). (a) Tube voltage (kV) versus breast thickness. (b) Tube load (mAs) versus breast thickness. (c) Tube voltage (kV) versus tube load (mAs).

The target/filter combination for FFDM was found to be Mo/Rh for breast thickness less than 4 cm (9.5%) and Rh/Rh for breast thickness larger than 4 cm (90.5%). For all DBT exposures, Rh/Rh combination was used regardless of breast thickness. The kV (Fig. 1A) and mAs (Fig. 1B) both increased with increasing breast thickness for FFDM and DBT techniques. Furthermore, the relationship, in Figure 1C, between kV and mAs indicated that the mAs increased with kV. Mammographic parameters such as kV, mAs and target/filter combination used according to breast thickness for FFDM and DBT techniques are summarised in Table 1 and Figure 1A–C.

## Radiation dose of FFDM and DBT

Both ESD and AGD were found to increase linearly with increasing breast thickness. However, the range of ESD and AGD values observed was smaller for DBT compared to FFDM. The highest median for ESD in mGy for FFDM and DBT techniques for the six different compressed breast thicknesses was (3.3, 3.1), (4.7, 4), (5.5, 4.7), (6.7, 5.9), (8.1, 7.8) and (9.1, 8.9), respectively. However, the median for total AGD in mGy for FFDM and DBT techniques for the six different compressed breast thicknesses was (3.3, 1.8), (4.5, 2.5), (4.6, 2.6), (5, 3), (5.6, 3.6) and (6, 4), respectively. The median and IQR for highest ESD and for total AGD of a single‐view acquisition for six different breast thicknesses categories are summarised in Table 2. The Kruskal–Wallis test with pairwise comparison of FFDM and DBT examinations of the whole population showed that both ESD and AGD were significantly lower for DBT (both *P* < 0.001). The Mann–Whitney test showed that there was a statistically significant difference in the median ESD and total AGD values between FFDM and DBT techniques (*P* <0.001). The Wilcoxon signed‐rank test showed that there was a significant difference in the median ESD and median total AGD values (*P* = 0.001) between both FFDM and DBT techniques.

**Table 2:** The median (IQR) (min, max) for highest ESD and total AGD in mGy for FFDM and DBT techniques for different compressed breast thicknesses. There was a statistically significant difference in the median ESD and total AGD values between FFDM and DBT techniques (*P* <0.001).

Technique Parameter Breast Thickness	FFDM		DBT		Difference (%)	
	Highest ESD‐mGy	Total AGD‐mGy	Highest ESD‐mGy	Total AGD‐mGy	ESD*	AGD^
2.1–3 cm	3.3 (1.3) (2.3–3.9)	3.3 (1.5) (2.8––4.2)	3.1 (0.5) (2.6–3.3)	1.8 (1.0) (1.5–3.1)	0.2 (6.1)	1.5 (45.5)
3.1–4 cm	4.7 (0.5) (3.4–5.3)	4.5 (1.3) (3.5–4.8)	4.0 (0.7) (2.8‐5.2)	2.5 (0.3) (1.9–3.0)	0.7 (14.9)	2.0 (44.4)
4.1–5 cm	5.5 (1.5) (3.6–8.5)	4.6 (1.6) (4.1–5.7)	4.7 (1.2) (2.8–9.3)	2.6 (0.5) (1.8–3.9)	0.8 (14.5)	2.0 (43.5)
5.1–6 cm	6.7 (1.4) (3.9–10.5)	5.0 (0.4) (4.1–8.3)	5.9 (1.8) (0.2–9.9)	3.0 (1.2) (2.1–4.8)	0.8 (12)	2.0 (40)
6.1–7 cm	8.1 (1.5) (5.5–11.1)	5.6 (0.5) (4.8–6.6)	7.8 (3.3) (5.1–16.2)	3.6 (1.1) (2.5–7.0)	0.3 (3.7)	2.0 (35.7)
7.1–8 cm	9.1 (1.5) (5.9–10.0)	6.0 (0.4) (4.7–6.3)	8.9 (2.9) (5.9–15.5)	4.0 (1.5) (3.0–6.2)	0.2 (2.2)	2.0 (33.3)

AGD = average glandular dose; DBT = digital breast tomosynthesis; ESD = entrance skin dose; FFDM = full‐field digital mammography.

* (ESD FFDM‐ ESD DBT/ESD FFDM) × 100.

^^^ (AGD FFDM‐AGD DBT/AGD FFDM) × 100.

It was observed that the percentage difference (%) between ESD values for both FFDM and DBT increased up to the breast thicknesses of 6 cm (6.1% to 12%) but thereafter, it decreased with increasing breast thickness (to 3.7% and to 2.2%). However, the percentage difference between total AGD values for FFDM and DBT was constant (45.5% to 33.3%). The percentage difference between the highest median ESD and median total AGD values of the six different breast thicknesses categories for FFDM and DBT techniques was summarised in Table 2.

**Figure 2:**
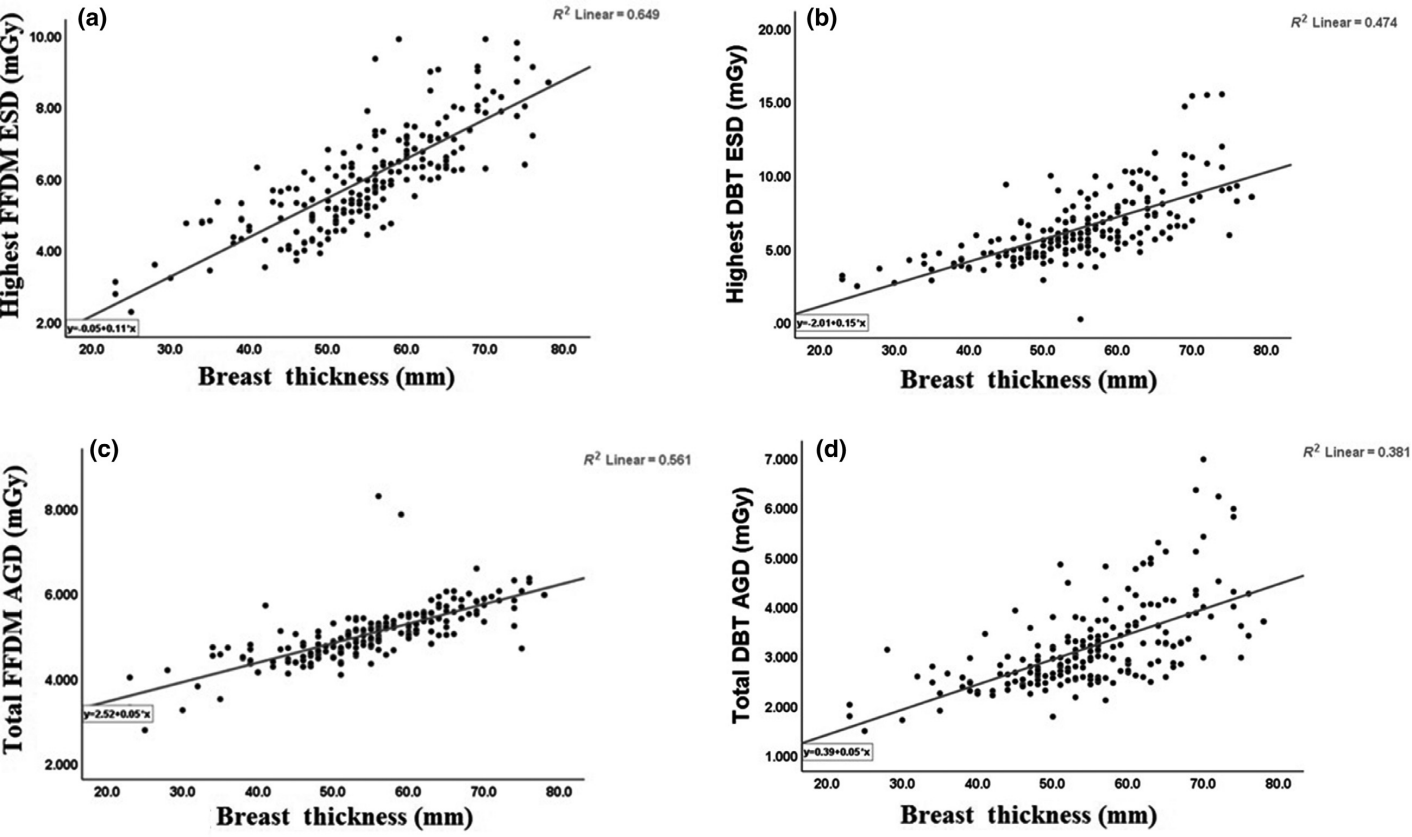
The highest entrance skin dose (ESD) and total average glandular dose (AGD) versus breast thickness for full‐field digital mammography (FFDM) (a and c) and digital breast tomosynthesis (DBT) (b and d).

The linear regression test showed a significant positive correlation between ESD and kV (*r* ^2^ = 0.31 and 0.49, *P* =0.001), mAs (*r* ^2^ = 0.44 and 0.68, *P* = 0.001), and breast thickness (*r* ^2^ = 0.56 and 0.47, *P* =* *0.001) for FFDM and DBT techniques, respectively. However, there were significant positive correlations between total AGD and kV (*r* ^2^ = 0.26 and 0.56, *P* = 0.001), mAs (*r* ^2^ = 0.62 and 0.79, *P* = 0.001), and breast thickness (*r* ^2^ = 0.56 and 0.38, *P* = 0.001) for FFDM and DBT techniques, respectively. The results are represented in Figure 2A–D.

Generally, in this patient population, the highest median of ESD and total AGD for FFDM was significantly higher than DBT technique (*P* = 0.001) for all the compressed breast thicknesses seen here.

## Discussion

In the current study, we used the radiation exposure from a commercially available mammography unit, where the main discerning feature was the step‐and‐shoot technique. In our study, all breasts were of fibro‐fatty nature with a majority of them (84%) being 4–7 cm thicknesses. The common kV/mAs value range of 28–32/50–56 with a target/filter material combination of Rh/Rh resulted in consistent clinical outcomes.

Since glandular tissue is the most radiosensitive part of the breast, the metric of choice to estimate breast dose is the AGD. The ESD was considered in our study for comparison purposes. In this study, the median of total AGD received by patients during a single‐view DBT acquisition was slightly lower compared to a two‐view FFDM acquisition. The difference was significant for ESD and AGD between FFDM and DBT. Importantly, for all acquisitions, the AGD was below the acceptable limits reported by the European Reference Organization for Quality Assured Breast Screening and Diagnostic Services guidelines.^16^ According to previous studies ^17^, ^18^ **,** there was a statistically significant difference between AGD derived from mammography machine and calculated AGD. Previous studies have published AGD dosimetric data for DBT of 1.74 mGy (*n* = 179) to 2.56 mGy (*n* = 300) ^18^, ^19^, ^20^, ^21^, slightly higher than in the present study (1–2 mGy/breast). The reported mean AGD values depend, both, on the vendor‐specific technical implementation to achieve an optimum between image quality and radiation dose^11^ as well as the breast thickness distribution of the population under study.

The low total AGD in DBT observed in the current study might be attributed to several reasons. First, the higher total AGD in FFDM can be attributed to the two views per breast in FFDM compared to one view per breast in DBT. Second, it might be partially explained by the use of a step‐and‐shoot technique in combination with a unique DBT anti‐scatter grid. Although the grid absorbs a part of the primary radiation, the scatter‐to‐primary ratio is substantially improved, thereby allowing the radiation dose to be lowered while maintaining good image quality.^22^, ^23^ Finally, a third factor that might have contributed to the observed lower total AGD in DBT than FFDM in our study was the use of Rh/Rh as a target/filter material combined with high tube voltages (29 kV and 31 kV) and lower tube loads (Table 1) implemented by the mammography unit manufacturer at the same compressed breast thickness.

All the non‐parametric statistical tests showed significant difference between ESD and AGD for FFDM and DBT techniques. Interestingly, the values of ESD and AGD were lower for DBT than FFDM technique. The results of this study are a promising development if DBT is to be considered as a breast cancer screening/detection tool. A review study indicated that the addition of DBT to a standard 2‐view DM significantly improved the accuracy as a result of a reduced number of false‐positive findings.^24^ Another study has indicated that single‐view DBT may potentially fully replace the conventional 2‐view DM.^11^, ^25^ For the current patient population, this would translate into a dose reduction of 2 mGy per breast per screening session. An additional benefit of single‐view DBT would be the reduction of patient discomfort as only one breast compression is needed per examination.

Our study has several limitations. First, in the breast imaging unit used in this study, the number of DBT examinations acquired was less than the number of FFDM. To enable a pairwise comparison of DBT and FFDM, only complementary examinations that were acquired on the same mammography unit were selected. Second, the diagnostic performance of DBT is not determined in our study because DBT examinations confirmed the clinical decision‐making based on the FFDM examinations by our experienced radiologists. Third, the data were not normally distributed, and all breast tissues were of fibro‐fatty composition. Fourth, our data were acquired from one GE mammography unit and therefore the DBT techniques used by other manufacturers with different equipment design may differ from the dose data presented here. Finally, our main limitation in this study was that we depended on radiation dose data directly derived from mammography unit. However, our quality assurance programme includes regular comparison of measured ESD values with those generated by the mammography unit. Those records show that the agreement between measured and unit generated ESD values were within measurement error margins. There are some studies that show small discrepancies, of the order of 0.2 mGy overestimation by GE units, between AGD reported by the mammography units and those calculated from measured ESD.^17^, ^18^ Since the AGD reported in this study are based on ESD and AGD reported by the unit, the values here could be slightly different. Further studies are needed with a larger sample size and different mammography units to explore which exposure factors produce the lowest radiation dose with acceptable image quality for FFDM or/and DBT techniques.

## Conclusions

The total AGD for a single‐view DBT was lower than a two‐view FFDM for single breast imaging. This offers opportunities for the implementation of DBT in the breast cancer detection with a consideration of clinical outcome and quality of diagnosis.

## Financial Information

No funding was requested or granted for this study.

## Conflict of Interests

The authors declare no conflicts of interest.
